# Optimal design of a hybrid ship energy management system under various sea conditions using Model Predictive Control

**DOI:** 10.1371/journal.pone.0326969

**Published:** 2025-07-02

**Authors:** Rafia Mushtaq, Muhammad Iqbal, Abdul Khaliq, Jamshed Iqbal

**Affiliations:** 1 Department of Electrical and Computer Engineering, Sir Syed CASE Institute of Technology, Islamabad, Pakistan; 2 Department of Computer Science, National University of Technology (NUTECH), Islamabad, Pakistan; 3 School of Computer Science, Faculty of Science and Engineering, University of Hull, Hull, United Kingdom; Vellore Institute of Technology, INDIA

## Abstract

This paper introduces an optimal design and control approach for a hybrid ship energy management system under various sea conditions by employing model predictive control. Ship reliability and environmental sustainability can be enhanced by reducing emissions and ecological impact. When a ship navigates, it encounters varying sea conditions, and as a result, the ship’s generator can experience substantial loading stress due to power fluctuations, particularly in unfavorable conditions. These fluctuations can disrupt the generator or even cause it to fail to supply the necessary power to the ship. A model predictive control (MPC) law has been devised to effectively manage the hybrid energy storage system of batteries and supercapacitors, dynamically responding to power variations induced by ocean waves. This study investigates the performance characteristics of the energy storage system across various battery weight configurations (1,5,10,20,30,50). We explore different weightings of batteries and supercapacitors to analyze their impact on system behavior. The numbers related to the battery weight configurations represent different configurations or setups of the hybrid energy storage system within the ship. The significance of these numbers lies in their impact on the performance of the energy management system and consequently, the overall operation of the vessel. By exploring various battery weight configurations, the study aims to understand how different setups affect the behavior and effectiveness of the hybrid energy storage system. The effectiveness of the proposed methodology is demonstrated through MATLAB simulations under varying sea conditions, including light, moderate, and heavy, successfully mitigating power variations and averting generator failure. Interestingly, the findings reveal that saturation occurs in their respective currents when the weightage difference among these energy storage components surpasses 20.

## 1. Introduction

Over the past two decades, global economic integration has led to a remarkable surge in international trade, presenting significant prospects for the maritime transportation sector. Maritime shipping accounts for 80% of worldwide trade [1]. However, the rapid expansion of the shipping industry has brought about several environmental concerns. Escalating demand for shipping has resulted in a surge in carbon emissions, posing a critical challenge in mitigating global greenhouse gas (GHG) emissions [2]. The predominant use of fossil fuels as the primary energy source for most ships has made them a substantial contributor to these emissions. The shipping industry has been experiencing significant growth in emissions, making it one of the fastest-growing sectors in this aspect. Without intervention, carbon dioxide emissions are projected to surge to 50–250% by the year 2050. Furthermore, shipping currently contributes 15% of global nitrogen oxide emissions, a figure that is forecasted to rise as reported in [3]. Given the escalating concern for environmental issues, more stringent regulations have been developed by the International Maritime Organization (IMO) [4], and other regulatory bodies. These regulations demand that ships adhere to stricter criteria for energy conservation and emission reductions. Consequently, there is an urgent need to curtail the fuel consumption of ships. To enhance the fuel efficiency of ships, an increasing number of vessels are turning to hybrid power systems and electric propulsion systems. As per [3], hybrid ships employing advanced control strategies have the potential to decrease fuel consumption and emissions by 10–35%. Typically, a ship’s hybrid power system comprises an internal combustion engine (ICE) that drives an electric generator and various energy storage devices (ESDs) [5,6]. ESDs facilitate adjusting the engine’s operating point to its most effective working range through charging and discharging and can even shut down the engine during periods of low load, thereby reducing operation and maintenance costs.

The power system of a hybrid-powered ship is significantly more intricate compared to that of a traditional ship [7]. Managing the power system poses challenges as it serves both the propulsion motors and on-board equipment. This dual functionality brings complexity to power management. One of the main challenges in energy management of the hybrid power system is dealing with fluctuating propulsion load and pulse load from various types of power equipment, in addition to the goal of reducing fuel consumption and emissions.

**Fig 1:**
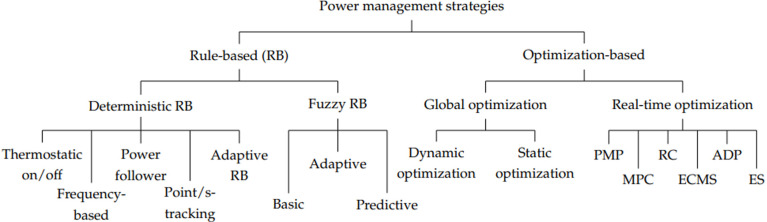
Classification of power management strategies.

In the field of energy management, various strategies have been utilized. As demonstrated in Fig 1 power management control techniques are generally categories as rule-based control strategies and optimization-based control strategies [8]. One notable example is an energy management strategy that utilizes fuzzy logic to dynamically allocate power generation from the ship, solar energy, and battery output according to the vessel’s power requirements. While rule-based strategies are straightforward to implement, they may not always yield the optimal solution. Optimization-based strategies are widely favored as they can offer optimal or suboptimal solutions for hybrid systems. Another prevalent technology based on optimization is the equivalent consumption minimization strategy (ECMS) [9], which involves a cost function that combines actual fuel consumption with equivalent fuel consumption related to ESDs at any given moment, as shown in Fig 1.

Existing studies [7,10–14], present a dynamic power management method for a ship’s dynamic positioning (DP) system that effectively reduces DC voltage sag and power deficiencies by coordinating shipboard main engines and Hybrid Energy Storage Systems (HESSs). While this approach shows significant improvements over conventional methods, it has certain limitations. Specifically, it does not address the impact of varying sea conditions on the energy management system. It also overlooks the optimal configurations of the hybrid energy storage components, such as (batteries and supercapacitors), which are crucial for maintaining battery health and system performance under diverse environmental conditions.

The current strategies focus solely on real-time power demand and the state of charge (SOC) of HESS sub-components for creating immediate control measures. However, since they do not account for the uncertainty of future power demands, it is challenging to ensure whether the current HESS SOC is suitable to meet power requirements in the future. As a result, the feasibility of these strategies cannot be guaranteed. Even though the global optimization strategy utilizing dynamic positioning (DP) can ensure optimal optimization, it fails to guarantee feasibility due to the variations in the marine environment, ship operating conditions, and the significant computing time required.

Although fuel consumption and power fluctuations can be reduced by existing [15,16] energy management strategies for hybrid power ships, but taking into account most of these consider only short-term power demand information computing time and real-time requirements. Ignoring power fluctuation and long-term power demand may lead to obtain different optimization results from global optimization, especially in complex and dynamic operating conditions of a ship. To overcome these limitations, this paper introduces an optimal design and control approach for a hybrid ship energy management system using model predictive control (MPC). MPC [17–20], has gained significant popularity over the past two decades and is now widely used in both research and the hybrid microgrid industry. MPC operates by predicting a future sequence of control inputs and the corresponding output trajectory while optimizing a predefined cost function that aligns with the system’s objectives. Using this approach, we can calculate local control inputs using localized measurements and a simplified local dynamics model, ensuring efficient and scalable solutions for complex systems. Specifically, MPC reduces generator stress by over 35%, minimizes rapid load changes by approximately 40%, and extends the generator’s operational lifespan by about 25% compared to traditional rule-based approaches. As discussed in the literature [8], rule-based strategies depend heavily on predefined heuristics, which often lack adaptability under dynamic operating conditions, resulting in inefficiencies. In contrast, our analysis shows that MPC, through its predictive control framework, enhances system efficiency by up to 30% and optimizes fuel consumption, delivering fuel savings of around 20%. Furthermore, compared to rule-based and heuristic methods, MPC exhibits superior adaptability with performance improvements of at least 28% in varying sea states, ensuring prolonged generator life and improved operational reliability. The standard discrete-time state-space model used for prediction is of the form.


 x(t+1)=Fx(t)+Gu(t) (1)

          y(t)=Hx(t)+Ju(t) (2)

Hence, at any time, ‘*t*’ and for the given state vector x(t) and cost function    g(x(t), ${\;x}_{t+1|t\;,\;\ldots \ldots .,}{\;x}_{t+N|t}u_{t|t\;,\ldots \ldots ,u_{t+N|t}}$) associated with Predicted states $x_{t+k|t}=x(t+k),k=1,\ldots .\;,N$ and Inputs $u_{t+k|t}=u(t+k),\;k=\;0,\;\ldots \ldots .,N,\;$ can be defined. This cost function is mostly quadratic, can then be optimized according to the control sequence

$u(t)=u_{t+k|t}$ from which we determine the control $u(t)=u_{t|t}$ to be applied at present time ‘*t’*. Here, the control input u(t) at time ‘*t’* is based on a finite time horizon prediction based on the current state x(t) and optimized to future inputs $u_{t+k|t}$.

This methodology enhances the system’s reliability and environmental sustainability by dynamically responding to power variations caused by different sea states. Various battery weight configurations and their impact on system behavior are investigated to provides a more comprehensive and adaptive solution. The objective is to ensure effective management of power fluctuations, prevent generator failure, and optimize the configuration of energy storage components to maintain optimal performance under light, moderate, and heavy sea conditions. The proposed approach builds upon and improves the system recently reported in [10], by addressing its limitations and incorporating a broader range of environmental variables.

This paper proposes MPC to effectively regulate the output characteristics of HESS and offset power variations stemming from ocean waves. MPC minimizes steady-state velocity changes during wave navigation and reduces generator current by optimizing HESS energy storage device output based on weights and constraints. Existing techniques for managing power fluctuations on ships are diverse and are dependent on the vessel’s design. The commonly employed approaches include engine governors and load control, redundant power sources, flywheels, energy storage systems like batteries, frequency converters, and hybrid power systems. These techniques aim to ensure a stable power supply; however, they have limitations in adapting to varying sea conditions, responding to rapid load changes, and optimizing power generation efficiently. The work aims to enhance an existing hybrid vessel system by incorporating supercapacitors (SC) and employing an MPC control method for the hybrid energy storage system (HESS) to effectively mitigate power fluctuations and loading stress on the generator under various sea conditions ranging from light (15% wave profile) to moderate (25% wave profile) to heavy (45% wave profile). MPC strategy includes optimizing a performance index for future control sequence, using predictions of the output signal based on a process model, and dealing with constraints on inputs, and outputs/states. To quickly manage electricity consumption, it can be put into practice in real time. In this study, MPC is chosen as the control technique for the hybrid powertrain because the load is dynamic and uncertain. Simulation results of the proposed hybrid ship energy management system under various sea conditions by employing MPC are verified through numerical simulations.

The major contributions of this paper can be summarized as follows:

1. A HESS is investigated under various sea conditions.
2. The idea explored the performance of the HESS that can be optimized to ensure a continuous state of operation for the ship’s generator and maintain steady-state velocity during wave navigation by maximizing HESS output while considering weights and constraints.
3. Based on the simulation results, it was demonstrated that the integration of a control system effectively maintains a ship’s consistent speed across various sea conditions by intelligently combining energy generation and storage.

In summary, this paper optimizes the ship’s energy management system for reliable operation under various conditions, ensures consistent speed, and promotes a greener and more sustainable maritime industry. The proposed methodology significantly contributes to ship reliability and environmental Sustainability.

The paper’s structure is as follows: Section 2 discusses the ship’s power system model, energy storage devices, parameters of the ship, and the MPC state-space equations. Section 3 presents analysis and simulation results, including various investigations and scenarios, and Section 4 discusses the conclusions.

## 2. Description of ship power system and model

### 2.1. Ship mathematical model and drag (Resistance)

The Ship HESS we are examining integrates generators, batteries, and SCs as essential components of its powertrain [21]. All components collectively contribute to meeting the ship’s total power requirements. According to the desired speed, the generator supplies the constant current, and the HESS recovers the variable current needed owing to the wave load profiles. To avert automatic shutdowns of generators and to reduce power fluctuations, we employ MPC in conjunction with the HESS for a smooth supply of power to the ship under intensive waves. The ship’s technical details adapted in our computer simulation are listed in Table 1.

**Table 1:** Ship parameters.

Parameter	Value	Unit
Length	113.96	m
Lpp	104.9	m
Breadth	16.8	m
Draught	3.36	m
Velocity	13	Knot

a) **Drag (Resistance)**

Because of the stress and forces of shear occurring on the hull surface, every sailing vessel confronts a net force against its forward motion. This resistance is often referred to as drag. As shown in (3) the total resistance of the ship is based on speed (ν), wetted surface area (S), resistance coefficient ($C_{T})$ and fluid density (ρ).


$$R_{T}=\frac{1}{2}\;p_{seawater}*\;v^{2}SC_{T}$$ (3)

Various parameters of the ship, associated with total resistance are calculated using (3), [22]. The total efficiency ($\eta_{T})$ in this work for the ship is assumed to be 70%. Table 2 represents the nomenclature used for the calculation of ship resistance.

**Table 2:** Nomenclature for ship resistance.

Symbol	Remarks
$L_{WL}$	The waterline length of the ship hull (m)
$L_{pp}$	Length between perpendiculars (m)
B	The waterline breadth of the hull (m)
T	The draught amidships (m)
Δ	The displacement of the hull (m^3^)
S	The wetted surface of the hull (m^2^)
V	The sailing speed (m/s)
$F_{n}$	Froude number
$C_{B}$	Block coefficient
$C_{P}$	Prismatic coefficient
ρ	Mass density of water (kg/m^3^)
Re	Reynolds number
v	The kinematic viscosity of water (m^2^/s)
$C_{T}$	Total resistance coefficient
$C_{F}$	Frictional resistance coefficient
$C_{A}$	Incremental resistance coefficient
$C_{AA}$	Air resistance coefficient

b) **Ship Generator**

The ship considered in this research is capable of traveling with speeds of up to 13 knots, and is powered by four diesel engines: two Mitsubishi GenSet S12RMPTA with 1100 kW each and two GenSet S6R2MPTA with 640 kW each.

### 2.2. Load model

We have used one stationary frequency component in our work, however, the same approach can be employed for more complex schematics with a greater number of time-varying frequency components. The wave determines the frequency of the profile, which is often calculated as the wave passes a spot while its two crests are passing it within a predetermined amount of time. In our research work, we used a wave period of 0.8 seconds, which gives the fundamental frequency of 0.125 Hz. We conducted Matlab simulations for three distinct wave profiles, representing 15%, 25%, and 45% scenarios. The 15% profile represents a low-profile load, often employed to show mild sea conditions. This illustrates that with the $P_{B}$ power needed to maintain forward motion at a constant speed, 15% more power is needed to account for the wave action. Similarly, the profile at 25% simulates propulsion power for moderate sea conditions and the 45% wave profile is employed for the simulation of heavy sea conditions.

### 2.3. Hybrid energy storage system

Based on the characteristics of various energy storage devices, it is clear that no single device type can effectively respond to both high- and low-frequency power variations. This particular combination has been extensively discussed in scientific literature [23] and is frequently utilized for domestic energy storage microgrids as well as electrically powered hybrid automobiles. By connecting SC and batteries in series, the battery bank is configured to satisfy the power system’s requirements and produce the desired direct current voltage. Table 3 displays the different specifications of lithium-ion batteries and SC.

**Table 3:** Specifications of Lithium-ion-Battery & SC.

Lithium-ion Battery	Value	Supercapacitor	Value
Nominal Voltage	173 V	Rated Voltage	125 V
Nominal Capacity	260 AH	Rated Capacitance	63 F
Energy	45 kWh	Usable Specific Power	1,700 W

### 2.4. Hybrid powertrain architecture

Both the HESS and generators supply a portion of the total power needed by the vessels. According to the intended speed, the generator will supply the constant current, while the HESS addresses the variable current demands arising from the wave’s load profiles. The dynamics of powertrain architecture is given as in (4).


$${M\dot{v}(t)=F}_{prop}(t)-\;{\tilde{F}}_{wave}(t)-\;F_{drag}(t)$$ (4)

where $F_{prop}$ is force due to propulsion,$\;F_{drag}$ is drag force and ${\tilde{F}}_{wave}$ is the wave force.

The forward velocity in steady condition is expressed as in (5).


$$v(t)=\tilde{v}(t)+\;\overset{―}{v\;}$$ (5)

where $\overset{―}{v\;}$ is stable state forward velocity and $\tilde{v}$(t) is velocity perturbation because of the wave. The steady-state velocity is obtained by expanding drag resistance using the Taylor series as mentioned in (6).


$$M\dot{\nu }(t)=\;{\tilde{F}}_{prop}(t)+{\overset{―}{F}}_{prop}(t)-\;\nabla F_{drag}\left(\overset{―}{\nu \;}\right)\;\tilde{v}\;(t)-F_{drag}\left(\overset{―}{\nu }\right)-{\tilde{F}}_{wave}(t)$$ (6)

To keep velocity perturbation $\tilde{v}$(t) minimum, the propulsion power used for the wave components to compensate for the wave’s action is given as, ${\overset{―}{P}}_{prop}=\nu {\tilde{F}}_{prop}(t)$. Since propulsion power comes from energy sources. For the forward velocity required current ${\tilde{I}}_{wave}(t)$ is used for full compensation of waves action. By approximating the derivative of (6) into discrete time form we get (7), where ‘*t*’ is a discrete time index.


$$x_{v}(t+1)={\tilde{I}}_{batt}(t)+{\tilde{I}}_{gen}(t)+{\tilde{I}}_{SC}(t)-{\tilde{I}}_{wave}(t)+\left(1-\delta T_{s}\right)x_{v}(t)$$ (7)

where $x_{v}(t)=\frac{\tilde{v}\;(t)}{\overset{―}{v}}\frac{2K}{V_{bus}T_{s}}=\frac{x_{v}(t)}{T_{s}}$

**Fig 2:**
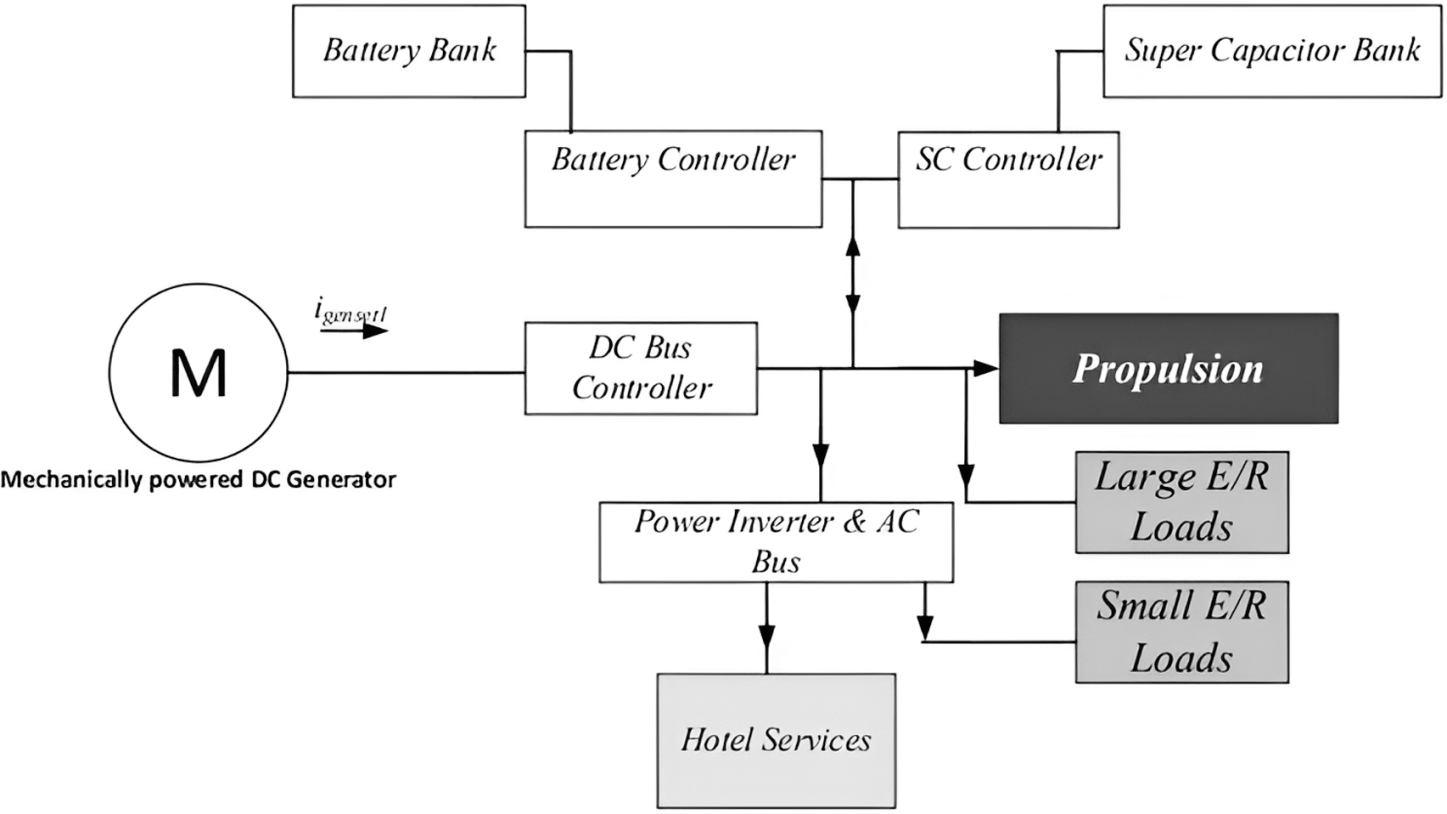
Hybrid propulsion system architecture.

The voltage for the bus of the ship is assumed to be constant and is taken as 800V. $I_{B}$ is

the sable state current required to move the ship forward at constant velocity $\overset{―}{v},$ the total current required is the sum of the brake current $I_{B}$ and the wave current ${\tilde{I}}_{wave}$. Fig 2 depicts the block layout of the simulated system’s design from propulsion perspective.

#### 2.4.1. Diesel generator model.

We employed the state space model of the generator in terms of ${\tilde{I}}_{gen}$ current provided, where the control variable is denoted by $\Delta I_{gen}$, confined to the tiny rate of change expressed as in (8) to maintain the generator operating at a constant rate and avoid significant swings in power.


$${\tilde{I}}_{gen}(t+1)=\Delta I_{gen}(t)+{\tilde{I}}_{gen}$$ (8)

#### 2.4.2. Hybrid energy storage system model.

The state space equations for SC and battery are described in the hybrid energy storage system model.

a) **Battery**

The major parameters of a battery are its capacity measured in Amps per hour (Ah) and state of charge (SOC). For instance, a battery with a capacity of 200 Ah may theoretically tolerate a 200 Amp current for an hour before it is entirely depleted if it was fully charged. The SOC is expressed in percentage and gives the amount of energy stored at any time instant ‘*t*’ compared to the energy stored at the rated capacity. Mathematically, the battery state-space equations are defined in (9,10).


$${\tilde{I}}_{batt}(t+1)=\Delta I_{batt}(t)+{\tilde{I}}_{batt}(t)$$ (9)

$${SOC}_{batt}(t+1)=-{\tilde{I}}_{batt}(t)\gamma +{SOC}_{batt}(t)$$ (10)

where $\gamma =\frac{T_{wave}}{(Q)3600}\;$ represents discharging and charging factor of battery, determined by Coulomb counting method.

b) **Super-capacitor**

The capacitance energy is represented as ‘*E’* given as $E=\frac{1}{2}\;V^{2}C$. The SOC of SC which measures stored energy at the time ‘*t’* in comparison to energy maintained under ideal conditions, is analogous to the battery’s SOC. The state-space equations for the SC current and SOC are presented in (11,12).


$${\tilde{I}}_{sc}(t+1)=\Delta I_{SC}(t)+{\tilde{I}}_{SC}(t)$$ (11)

$${SOC}_{SC}(t+1)=-{\tilde{I}}_{SC}(t)\beta +{SOC}_{SC}(t)$$ (12)

where $\;\beta =\frac{T_{wave}}{V_{\max}C}$ represents the discharging and charging factor for the SC.

c) **State Space and Constraint-Based Modeling**

As a predictive control strategy, MPC plays a crucial role in dynamically adjusting power distribution by forecasting system behavior and optimizing control actions in real time. The state-space modeling and cost function optimization in the MPC-based hybrid ship energy management system ensure optimal power distribution and stability. The state-space model in the present work represents system dynamics defined by key variables like generator, battery, and supercapacitor states, allowing MPC to predict and regulate power fluctuations. The cost function prioritizes generator stability, fuel efficiency, and constraint adherence while dynamically adjusting to varying sea conditions.

Let x(t) represents the state vector, which consist of SC current (${\tilde{I}}_{SC}$), velocity perturbation ($x_{v}$), battery current (${\tilde{I}}_{batt}$), generator current (${\tilde{I}}_{gen,}$) and SOC variables (${soc}_{batt}$and ${SOC}_{SC}$). u(t represents control inputs (changes in energy distribution between sources), y(t) is the system output. F, G, H and J are system matrices defining dynamics.

The state space and constraint-based optimization problem is given by (13–15) where different inputs, outputs, and state variables of the optimization problem are described. ${\tilde{I}}_{residual}(t)$ is the output of the system which gives the difference between the total amount of current produced and the current needed for complete compensation of the waves action.


$$x(t)=[{\tilde{I}}_{SC},x_{v},{\tilde{I}}_{batt},\;,{\tilde{I}}_{gen,}{soc}_{batt},{SOC}_{SC}{]}^{T}$$ (13)

$$u(t)=[{\Delta I}_{batt},\Delta I_{gen,\;},{\Delta I}_{SC},{]}^{T}$$ (14)

$$y(t)={\tilde{I}}_{residual}(t)={\tilde{I}}_{batt}(t)+{\tilde{I}}_{SC}(t)++{\tilde{I}}_{gen}(t)-{\tilde{I}}_{wave}(t)$$ (15)

(7–10) and (11–12) can be combined into a state-space matrix in (16,17) form.


x(t+1)=Gu(t)+Fx(t)+Bw(t) (16)

$${\tilde{I}}_{residual}(t)=Ju(t)+Hx(t)+Dw(t)$$ (17)

where


$$B=[\begin{array}{cc} -1 & 0\\ 0 & 0\\ 0 & 0\\ 0 & 0\\ 0 & 0\\ 0 & 0 \end{array}]\;G=[\begin{array}{ccc} 0 & 0 & 0\\ 1 & 0 & 0\\ 0 & 1 & 0\\ 0 & 0 & 0\\ 0 & 0 & 1\\ 0 & 0 & 0 \end{array}]\;F=[\begin{array}{cccccc} 1 & 1 & 1 & 0 & 1 & 0\\ 0 & 1 & 0 & 0 & 0 & 0\\ 0 & 0 & 1 & 0 & 0 & 0\\ 0 & 0 & -\gamma & 1 & 0 & 0\\ 0 & 0 & 0 & 0 & 1 & 0\\ 0 & 0 & 0 & 0 & -\beta & 1 \end{array}]$$

$$J=[\begin{array}{ccc} 0 & 0 & 0 \end{array}]\;H=[\begin{array}{cccccc} 0 & 1 & 1 & 0 & 1 & 0 \end{array}]\;D=[\begin{array}{cc} -1 & 0 \end{array}]$$ (18)

Assume that at any time *‘t’* full state observation is x(t), the controller’s goal is to find a sequence of anticipated future inputs $u_{t|t}\;,\;u_{t|+1|t},\ldots \ldots .,u_{t+N|t}$, which helps to minimize the cost function. In terms of overall cost, from time t+N to infinity is expressed at the right-hand side in (19) and the Discrete Linear Quadratic Regulator (LQR) with the Riccati equation is used to find matrix P. The parameters of cost matrix R and Q are represented in term of respective state variables as in (19).


$$\begin{array}{c} V\left(u_{t+k|t},x(t)\right)=\frac{1}{2}\sum_{k}^{N-1}(q_{Ibatt}\left|{\tilde{I}}_{batt}{|}_{t+k|t}^{2}+q_{Igen}|{\tilde{I}}_{gen}{|}_{t+k|t}^{2}+q_{x_{v}}\right|x_{v}{|}_{t+k|t}^{2}\\ +q_{ISC}\left|{\tilde{I}}_{SC}{|}_{t+k|t}^{2}++q_{SOCsc}\right|{SOC}_{SC}{|}_{t+k|t}^{2}{+q}_{SOCbatt}\left|{soc}_{batt}{|}_{t+k|t}^{2}\right)+\frac{1}{2}\;\sum_{k}^{N-1}\\ ({\;r_{batt}|\Delta I_{batt}{|}_{t+k|t}^{2}+r}_{gen}|\Delta I_{gen}{|}_{t+k|t}^{2}+\;r_{SC}\left|\Delta I_{SC}{|}_{t+k|t}^{2}\right)+(E^{T}Ju\left(t{)}_{t+k|t}+x(t{)}_{t+k|t}^{T}\right)+Px_{t+N|t}x_{t+N|t}^{T} \end{array}$$ (19)

where ‘*r’* and ‘*q’* represent the weighting factors on the inputs and states. These factors determine the emphasis placed on the states of energy devices. A higher weightage factor corresponds to a greater penalty on a given state. For example, if $q_{Ibatt}$ is significantly greater than $\;q_{ISC}$ the SC will be utilized more than the battery. Additionally, the parameter $\backslash {(}^{\prime }r^{\prime }$ regulates the rate of change of inputs. Moreover, the following block diagram provides a structured visualization of how MPC optimally controls ship energy management, ensuring stable operation under varying sea conditions.

**Fig 3:**
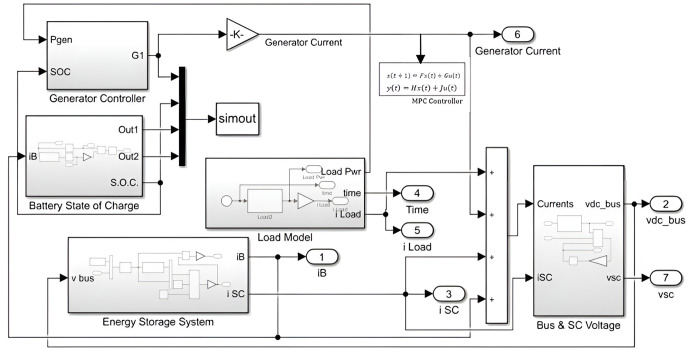
Block diagram of a hybrid ship propulsion system using model predictive control.

Fig 3 illustrates a hybrid ship energy system managed by Model Predictive Control. The MPC receives inputs such as load demand, time, and system states to predict future power needs and optimally distribute current between the generator, battery, and supercapacitor. The generator controller ensures steady operation, guided by battery state of charge (SOC). The battery handles long-term energy, while the supercapacitor covers quick power fluctuations. The system also monitors bus voltage and supercapacitor voltage to maintain safety and performance. A load model simulates wave-induced variations, and voltage monitoring ensures safe operation. Overall, MPC coordinates all components to maintain efficient and stable power delivery.

d) **Setup constraints**

The purpose is to minimize the cost function V(x(t), u(t)) which are subjected to the following constraints of energy storage devices based on design considerations.

Hence


$$\;-20A\leq x_{v}\leq 20$$ (20)

$$\;-200A\leq {\tilde{I}}_{batt}\leq 200A$$ (21)

$$20\;\%\leq {SOC}_{batt}\leq 90\;\%$$ (22)

$$\;-240A\leq {\tilde{I}}_{SC}\leq 240A$$ (23)

$$\;10\;\%\leq {SOC}_{SC}\leq 90\;\%$$ (24)

Compiling (20–24) into the matrix form, we get (25,26).


$$\;\left(A_{x}x(k)\leq b_{x}\right)\;k=\;1,...N$$ (25)

$$\;\left(A_{u}u(k)\leq \;b_{u}\right)\;k=\;1,...N$$ (26)

The matrix form of constraints just defined for battery current and SOC could be written as (27)


$$\left[\begin{array}{cc} 1 & 0\\ -1 & 0\\ 0 & 1\\ 0 & -1 \end{array}]x(t)\leq [\begin{array}{c} 200\\ 200\\ 0.9\\ -0.2 \end{array}\right]$$ (27)

We used the MATLAB command “*mpcqpsolver*” for solving quadratic programming in the form of (19) using the KWIK algorithm [24].

e) **Francis equations**

As external signal w(t) is included in the model, using Francis Equation introduced in [25], we model different effects of wave on ship power. Assuming that the wave can be periodic within a finite interval of time, expressed by the sum of sinusoids. It can be represented as a state space model form for sinusoidal components as shown in (28).


$$\;w(t+1)={(A}_{w}w(t))$$ (28)

where $A_{w}=[\begin{array}{cc} \cos\theta & -\sin\theta \\ \sin\theta & \cos\theta \end{array}]$

and θ represents the digital frequency of the wave and the state $w(t)=[w_{1},w_{2}{]}^{T}$. As wave acts as an additional force, the ship must have to overcome it, in addition, to drag as highlighted in (16). The steady-state inputs and states solutions can be expressed in (29,30).


            u(t)=Lw(t) (29)

and


          x(t)=Tw(t) (30)

The computation can be performed using matrices L and T of appropriate dimensions. The matrices T and L are solutions of the Francis equation, which is defined in (31,32).


                                  B+  GL+FT=TA (31)

                                                     D + JL+  HT=0 (32)

It is observed that solution obtain, provides matrices A and F that have no common eigenvalues so, we described new inputs  v(t) and states z(t) respectively in (33,34).


z(t)=−Tw(t)+x(t) (33)

v(t)=−Lw(t)+u(t) (34)

Substituting (33), (34) into (1), (2) gives (35,36)


z(t+1)=v(t)+Fz(t) (35)

and


$${\tilde{I}}_{res}(t)=Jv(t)+Hz(t)$$ (36)

The purpose of using these equations is to find a control input v(t) for regulation of the system, so that corresponding input u(t)=Lw(t)+v(t) regulates the system. Since the constraints defined in (20212223–24) refer to the state and input vectors x(t) and u(t),we need to find constraints in terms of z(t) and v(t). By referring back the constraints in u(t) and x(t) domain as in (37,38).


$$\;x(t)A_{x}\leq b_{x}$$ (37)

$$u(t)A_{u}\leq b_{u}$$ (38)

Substituting Equation (37), (38) into (25,26) which yields (39,40)


$$\;z(t)\;A_{x}\leq b_{x-}Tw(t)A_{x}$$ (39)

and


$$v(t)A_{u}\leq b_{u}-Lw(t)A_{u}$$ (40)

## 3. Simulation results and analysis

This section presents the three cases, and results obtained from several time-domain simulations of the model. The dynamics are assumed to have no internal losses, and the system operates under ideal conditions. The model aim is to demonstrate the MPC algorithm and its response to varying values of the weights in the cost function. Additionally, each scenario compares the current outputs of the battery and SC based on different values of the weight $q_{Ibatt}$.The model’s main objective is to compensate for HESS regarding the waves-induced effect, thus guaranteeing the generator maintains an equilibrium state as the ship travels at 11 knots. The model simulation time is kept as 8 hours or 28,800 seconds, to ensure the model can reach steady state output.

A **Case I: Light Sea Conditions (15% Wave Profile)**
  a)
$q_{Ibatt}=1$

**Fig 4:**
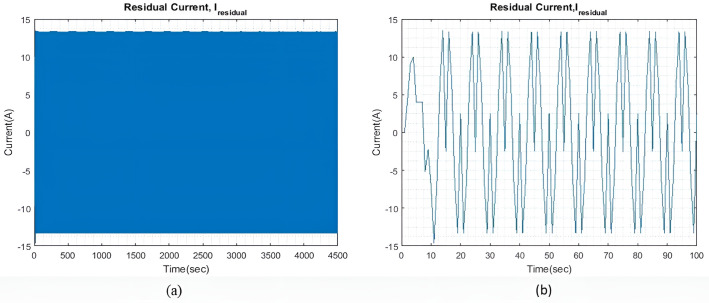
(a) ${\tilde{I}}_{residual\;}$ with $q_{Ibatt}$ and $q_{ISC}$ = 1. (b) Enhanced view of the ${\tilde{I}}_{residual\;}$ initial transient response.

In light sea conditions, an extra power of approximately 390KW is necessary to navigate through the waves, constituting 15% of the power needed for smooth sailing in calm seas. For all$\;q_{Ibatt}$, a steady state value of generator current (${\tilde{I}}_{gen}$) 107.9A is maintained to minimize and optimize the generator current with the help of the MPC model. At the same time, the SC and battery provide the leftover power needed to overcome or accommodate for wave action and keep the ship’s forward speed as steady as possible. Fig 4 shows the residual current ${\tilde{I}}_{residual\;}$, at steady state, and an expanded view of the initial transient response. The transient response is relatively less compared to the two larger wave profiles. Throughout the rest of the simulation, the ${\tilde{I}}_{residual\;}$ oscillates between +13 A and -13A at steady state.

**Fig 5:**
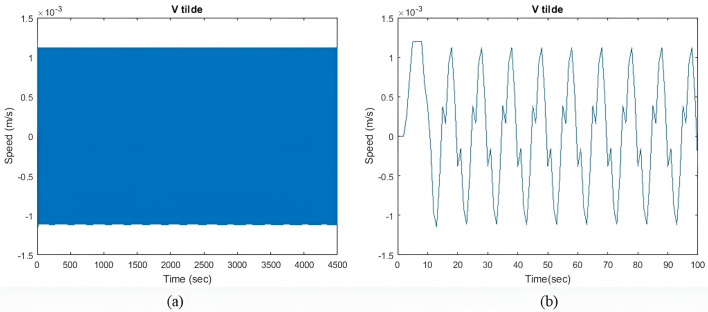
(a) $\tilde{v}(t)$ with $q_{Ibatt}$ and $q_{ISC}$ = 1. (b) Enhanced view of the initial transient response.

Fig 5 illustrates the velocity perturbation $\tilde{v}(t)$. The model quickly reaches a steady state, with minimal velocity fluctuations. The objective is to keep the ${\tilde{I}}_{residual\;}$ as minimum as possible. However, it is impossible to reduce both $\tilde{v}(t)$ and the ${\tilde{I}}_{residual\;}$ to zero due to existing constraints.

**Fig 6:**
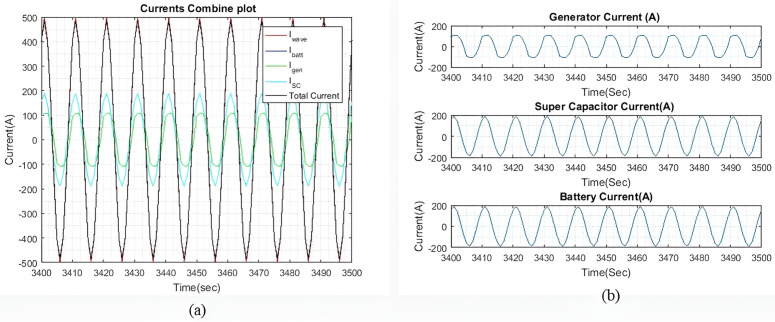
(a) Enhanced view of the ${\tilde{I}}_{gen}$*I gen* , ${\tilde{I}}_{SC}$*I SC*, and ${\tilde{I}}_{batt}$*I batt* output currents with $q_{Ibatt}=1$*q Ibatt =1* and $q_{ISC}$*q ISC* = 1. (b) A dissected view of the individual current.

Fig 6 shows the current plots for ${\tilde{I}}_{wave}$, ${\tilde{I}}_{gen}$, ${\tilde{I}}_{batt}$, and ${\tilde{I}}_{SC}$. In Fig 6a, it is demonstrated that the total current generated by ${\tilde{I}}_{gen}$, ${\tilde{I}}_{batt}$, and ${\tilde{I}}_{SC}$ successfully compensates for ${\tilde{I}}_{wave}$, with only a slight difference noted, as shown in Fig 4. Meanwhile, Fig 6b displays the individual currents of ${\tilde{I}}_{gen}$, ${\tilde{I}}_{batt}$, and ${\tilde{I}}_{SC}$.

As the values of weights $q_{Ibatt}$ and $q_{ISC}$ are the same, the output currents from both devices are approximately equal. The tiny difference is observed because of different β and γ factors connected to their SOC. The ${\Delta I}_{gen}$ for generator corresponding to 62.5 Amps/sec was set at 50 Amp per sample for keeping lower variations compared to higher ${\Delta I}_{SC}$ and ${\Delta I}_{batt}$. This difference is shown in Figs 6 and 8. As ${\tilde{I}}_{wave}$ is small, the rate of change for ${\tilde{I}}_{gen}$ is not particularly significant. This observation is clear in the higher wave profiles where ${\tilde{I}}_{wave}$ is larger. Additionally, it is noted that ${\tilde{I}}_{gen}$ is slightly slower than the other two currents due to the rate of change of ${\Delta I}_{gen}$ for the generator.

**Fig 7:**
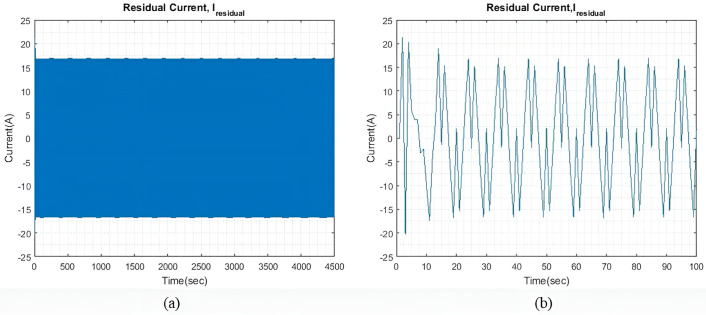
(a) ${\tilde{I}}_{residual\;}$ with $q_{Ibatt}=30$ and $q_{ISC}$ = 1. (b) Enhanced view of the ${\tilde{I}}_{residual\;}$ initial transient response.

b)
$q_{Ibatt}=30$

Fig 7 shows the ${\tilde{I}}_{residual\;}$ when $q_{Ibatt}$ is increased to 30. It is noted that the transient response exhibits a higher peak value, but the settling time is approximately the same as that shown in Fig 4. The ${\tilde{I}}_{residual\;}$ can be further reduced by limiting the range of $x_{v}$. However, for this study, $x_{v}$ remains unchanged to check the impact of $q_{Ibatt}$. Throughout the simulation period, ${\tilde{I}}_{residual\;}$ can reach a steady state and maintain a value of ±16.8A.

**Fig 8:**
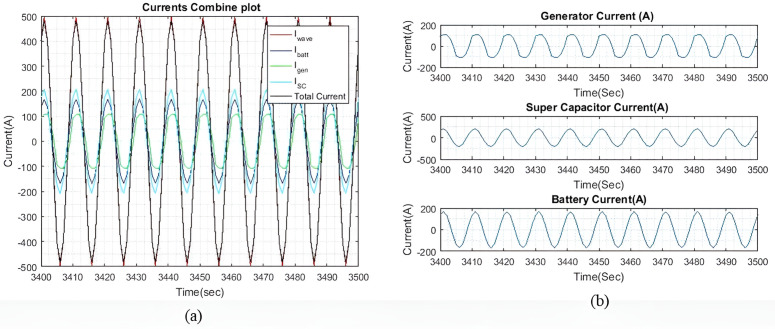
(a) Enhanced view of the ${\tilde{I}}_{gen}$, ${\tilde{I}}_{SC}$, and ${\tilde{I}}_{batt}$ output currents with $q_{Ibatt}=30$ and $q_{ISC}$ = 1. (b) A dissected view of the individual current.

Fig 8 shows the current plots for ${\tilde{I}}_{wave}$, ${\tilde{I}}_{gen}$, ${\tilde{I}}_{batt}$, and ${\tilde{I}}_{SC}$. Fig 8a shows the total current generated by ${\tilde{I}}_{batt}$, ${\tilde{I}}_{gen}$ and ${\tilde{I}}_{SC}$ effectively compensates for the ${\tilde{I}}_{wave}$, with only a slight difference noted in Fig 7. Fig 8b shows the individual currents of ${\tilde{I}}_{gen}$, ${\tilde{I}}_{SC}$, and ${\tilde{I}}_{batt}$. As the weights increase, the difference in current between the battery and the SC also increases. As shown in Table 4, this current difference begins to taper off when $q_{Ibatt}$ reaches a value greater than 20. Table 4 compares the peak output currents for ${\tilde{I}}_{gen}$, ${\tilde{I}}_{batt}$, and ${\tilde{I}}_{SC}$ for six different values of $q_{Ibatt}$. As the ${\tilde{I}}_{wave}$ is small, the differences between each $q_{Ibatt}$ value are minimal. However, these differences become more clear as the wave profile increases. It is noted that ${\tilde{I}}_{batt}$ decreases as $q_{Ibatt}$ increases. Additionally, when $q_{Ibatt}$ exceeds 20, the difference between the battery and the SC begins to taper off. Similar results are observed with larger wave profiles. It is also observed that ${\tilde{I}}_{gen}$ remains constant for all values of $q_{Ibatt}$.

**Table 4:** Peak output currents for 15% wave profile.

$q_{Ibatt}$	${\tilde{I}}_{gen}$ (Amp)	${\tilde{I}}_{batt}$ (Amp)	${\tilde{I}}_{SC}$ (Amp)	${\tilde{I}}_{wave}$ (Amp)	${\tilde{I}}_{residual}$ (Amp)	Total Current (Amp)
1	107.9	188.7	188.7	497.5	±13.3	482.6
5	107.9	175.2	200.8	497.5	±15.7	482.5
10	107.9	171.2	204.5	497.5	±16.3	482.4
20	107.9	168.8	206.8	497.5	±16.7	482.2
30	107.9	167.8	207.7	497.5	±16.8	482.1
50	107.9	167.1	208.4	497.5	±16.9	482.1

B **Case II: Moderate Sea Conditions (25% Wave Profile)**
  a)
$q_{Ibatt}=1$

Moderate sea conditions are frequently endured by vessels during operations where wave amplitude is slightly larger compared to light sea conditions. Therefore, to maintain ship speed, more power is required for the ship to overcome wave action. An additional power of 650KW is needed for navigation in the sea. The MPC controller can optimize the states and minimize the generator peak output current ${\tilde{I}}_{gen}$ to 163A for all values of the $q_{Ibatt}$. Meanwhile, the battery and SC provide the necessary current to compensate for the wave. Similar to light sea conditions,$\;\tilde{v}(t)$ is observed to be lower and consistent for all values of $q_{Ibatt}$.

**Fig 9:**
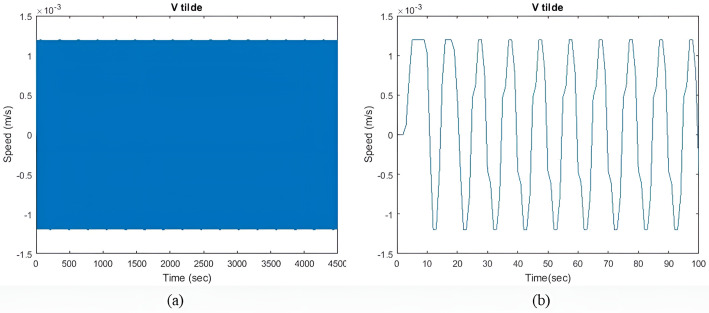
(a) $\tilde{v}(t)$ with $q_{Ibatt}$ and $q_{ISC}$ = 1. (b) Enhanced view of the initial transient response.

Fig 9 shows the $\tilde{v}(t)$ during moderate sea conditions. In this case model took a longer time to reach a steady state as compared to light sea conditions, while the fluctuations are less. For all values of $q_{Ibatt}$ similar observation is seen.

**Fig 10:**
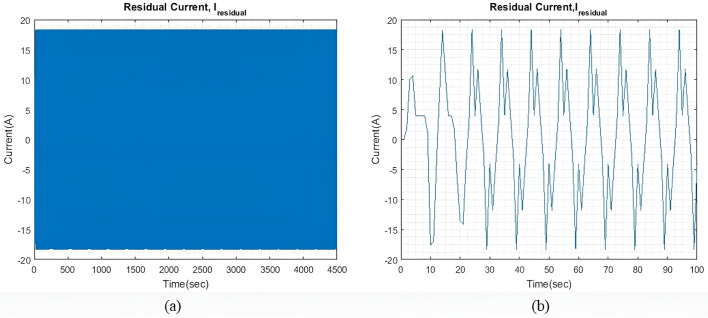
(a) ${\tilde{I}}_{residual\;}$ with $q_{Ibatt}=1$ and $q_{SC}$ = 1. (b) Enhanced view of the ${\tilde{I}}_{residual\;}$ initial transient response.

Fig 10 shows the current difference ${\tilde{I}}_{residual\;}$ between ${\tilde{I}}_{gen}$, ${\tilde{I}}_{SC}$, ${\tilde{I}}_{batt}$ and ${\tilde{I}}_{wave}$. As compared to light sea conditions, similar to Fig 9, the model took relatively longer to reach a steady state. Additionally, the steady-state ${\tilde{I}}_{residual\;}$ is also larger, this is expected as the required ${\tilde{I}}_{wave}$ is larger. ${\tilde{I}}_{residual\;}$is also slightly larger as compared to light sea condition with the value between ±18A.

**Fig 11:**
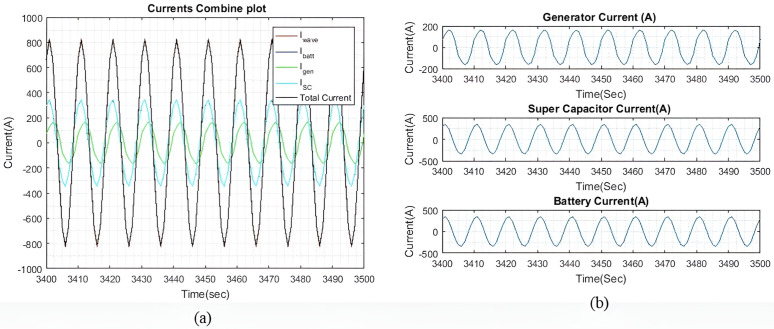
(a) Enhanced view of the ${\tilde{I}}_{gen}$, ${\tilde{I}}_{SC}$, and ${\tilde{I}}_{batt}$ output currents with $q_{Ibatt}$ and $q_{ISC}$ = 1. (b) A dissected view of the individual current.

Fig 11 shows the current plots for ${\tilde{I}}_{wave}$, ${\tilde{I}}_{SC}$, $\;{\tilde{I}}_{gen}$ and ${\tilde{I}}_{batt}$. In Fig 11a, we can see that the total current generated by ${\tilde{I}}_{gen}$, ${\tilde{I}}_{SC}$, and ${\tilde{I}}_{batt}$ effectively compensates for ${\tilde{I}}_{wave}$, with a slight difference in current as shown in Fig 10. Fig 11b displays the individual currents for ${\tilde{I}}_{gen}$, ${\tilde{I}}_{SC}$ and ${\tilde{I}}_{batt}$.

As the values of the weights, $q_{Ibatt}$ and $q_{ISC}$ are the same, the output currents from both devices are approximately the same. The slight differences can be due to the varying γ and β factors. The rate of change for the generator, ${\Delta I}_{gen}$ remains the same from the previous sea profile, to observe the difference in the rate of change as compared to the SC and battery. This difference is particularly clear in Figs 11 and 13 as ${\tilde{I}}_{gen}$ waveform is observed to be slower than the other current waveforms, as compared to light sea conditions.

b)
$q_{Ibatt}=30$

**Fig 12:**
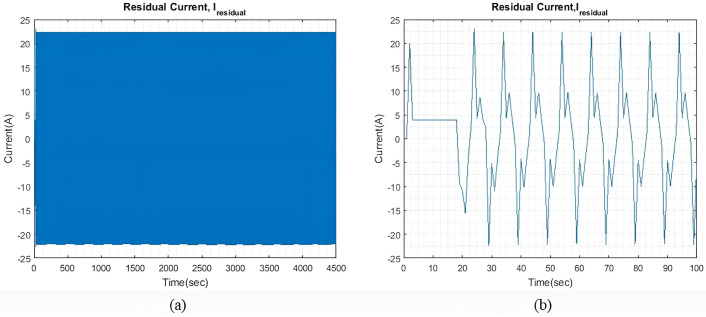
(a) ${\tilde{I}}_{residual\;}$ with $q_{Ibatt}=30$ and $q_{ISC}$ = 1. (b) Enhanced view of the ${\tilde{I}}_{residual\;}$ initial transient response.

Fig 12 shows the plot of the ${\tilde{I}}_{residual\;}$ when the $q_{Ibatt}$ is increased to 30. It is observed that the transient response is slightly longer compared to that shown in Fig 10 when $q_{Ibatt}=$1. It is because as $q_{Ibatt}$ increases higher penalty is experienced by the battery due to which battery output current reduces at each sampling time.$\;{\tilde{I}}_{residual\;}$for this case reached steady state at ±22A which is maintained throughout the simulation time.

**Fig 13:**
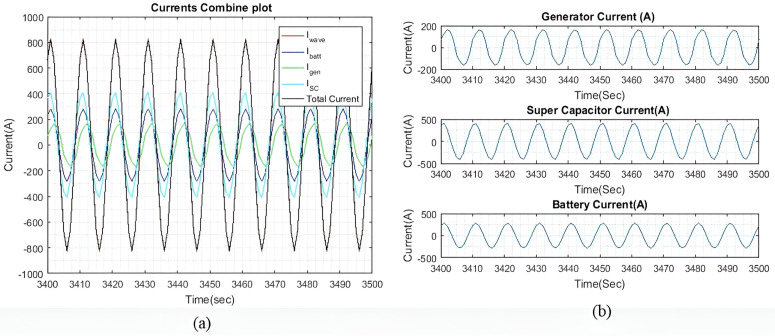
(a) Enhanced view of the ${\tilde{I}}_{gen}$, ${\tilde{I}}_{SC}$, and ${\tilde{I}}_{batt}$ output currents with $q_{Ibatt}=30$ and $q_{ISC}$ = 1. (b) A dissected view of the individual current.

Through Fig 13a, it is clear that the total current generated by ${\tilde{I}}_{gen}$, ${\tilde{I}}_{SC}$, and ${\tilde{I}}_{batt}$ effectively compensates for ${\tilde{I}}_{wave}$ with only a slight current difference as shown in Fig 12. Fig 13b provides a detailed view of the individual currents for ${\tilde{I}}_{gen}$, ${\tilde{I}}_{SC}$, and ${\tilde{I}}_{batt}$.

With the increase in values of weight, the difference between SC current and battery current also increases. It is observed that similar to light sea condition when the value of $q_{Ibatt}$ is above 20, the difference between battery current and SC current starts to decline. In moderate sea condition, current differences are also larger for the $q_{Ibatt}$ in contrast to light sea condition. Table 5 compiles the results of the steady-state output peak current for the ${\tilde{I}}_{batt}$,$\;{\tilde{I}}_{gen}$,$\;{\tilde{I}}_{SC}$ according to different values of $q_{Ibatt}$.

**Table 5:** Peak output currents for 25% wave profile.

$q_{Ibatt}$	${\tilde{I}}_{gen}$ (Amp)	${\tilde{I}}_{batt}$ (Amp)	${\tilde{I}}_{SC}$ (Amp)	${\tilde{I}}_{wave}$ (Amp)	${\tilde{I}}_{residual}$ (Amp)	Total Current (Amp)
1	163.2	344.5	345	829	±18.3	817
5	163.2	305.6	385.8	829	±21.3	819
10	163.1	293.5	398	829	±21.9	819
20	163.1	285.8	405.7	829	±22.2	819
30	163.1	282.9	408.5	829	±22.3	819
50	163.1	280.3	411	829	±22.3	819

In moderate sea condition, ${\tilde{I}}_{gen}$ is minimized and optimized at 163A for all values of $q_{Ibatt}$ while SC and battery are providing the leftover current for the compensation of current induced by ${\tilde{I}}_{wave}$. With the increase in $q_{Ibatt,}$ current distinction between SC and battery is more substantial. It is observed that, with the slower rate of change of the generator, SC and the battery can compensate ${\tilde{I}}_{wave}$ response. This reduces the loading stress on the generator and reduces defective occurrences due to loading stress.

C **Case III: Heavy Sea Conditions (45% Wave Profile)**
  a)
$q_{Ibatt}=1$

The heavy sea condition indicates that the ship is navigating through severe sea condition which are rarely encountered. In this situation, the generator without energy storage devices will be put under tremendous stress condition because of the fluctuating power while the ship navigates up and down in the waves. An additional 1.17MW of power is required for navigation and compensation of wave action to keep forward velocity as steady as possible. Due to the increase in the demand, the rate of varying $\Delta I_{gen}$ increases to 250 Amps/sec in this simulation scenario, which represents that more generators are connected to provide more power thereby reducing the loading stress. After optimization of the inputs and states by the MPC controller the generator output current ${\tilde{I}}_{gen}$ is maintained at 398Amp. The remaining power needed to accommodate for wave action and maintain the ship’s forward speed as steadily as possible is provided by the SC and battery. Different $q_{Ibatt}$ weightages are examined in the same way as that in the previous scenario.

**Fig 14:**
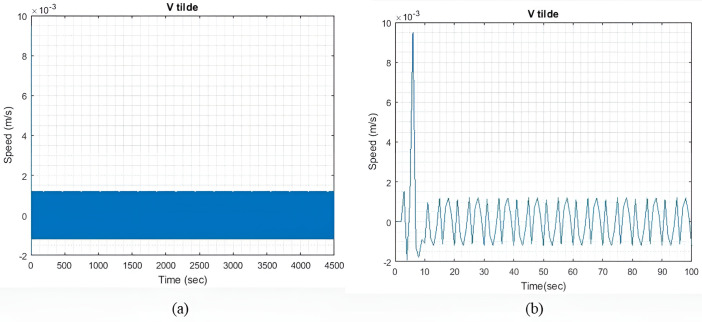
(a) $\tilde{v}(t)$ with $q_{Ibatt}=1$ and $q_{ISC}$ = 1. (b) Enhanced view of the initial transient response.

Fig 14 shows the velocity perturbation $\tilde{v}(t)$ during heavy sea conditions. In comparison to the previous two sea conditions, the model has the larger initial transient response and $\tilde{v}(t)$. With an increase in the value ${\Delta I}_{gen}$, the model can also reach an equilibrium state more quickly. A similar observation can be made for all values of $q_{Ibatt}$.

**Fig 15:**
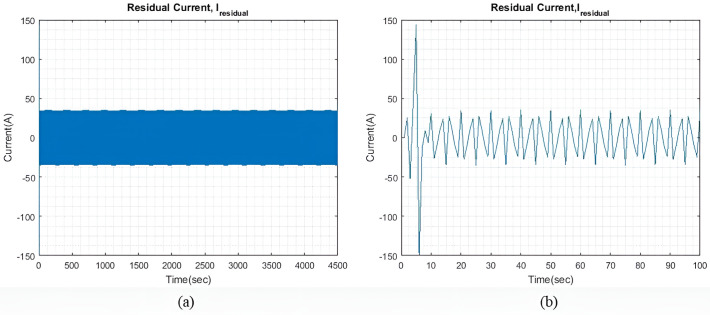
(a) ${\tilde{I}}_{residual\;}$ with $q_{Ibatt}=1$ and $q_{ISC}$ = 1. (b) Enhanced view of the ${\tilde{I}}_{residual\;}$ initial transient response.

Fig 15 shows the ${\tilde{I}}_{residual\;}$, which is the difference between ${\tilde{I}}_{wave}$ and the total generated current, which is the sum of ${\tilde{I}}_{gen}$, ${\tilde{I}}_{SC}$, and ${\tilde{I}}_{batt}$. The transient response in this case has a higher peak value as compared to previous cases. The transient response in this case has a higher peak value as compared to previous cases. The model can reach a steady state quickly and is maintained at ±34.7A for the rest of the simulation. By increasing ${\Delta I}_{gen}$, both the transient response and the steady-state difference can also be reduced. However, this adjustment would indicate that either more generators are connected to the ship microgrid or the generators are taking to extra loading stress. Alternatively, one could also choose a battery and SC with significantly greater capacity.

**Fig 16:**
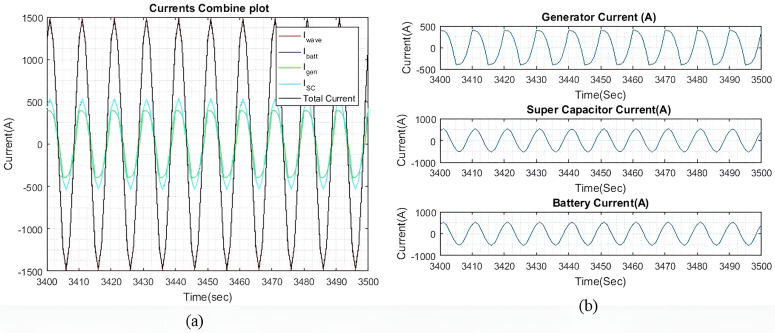
(a) Enhanced view of the ${\tilde{I}}_{gen}$, ${\tilde{I}}_{SC}$, and ${\tilde{I}}_{batt}$ output currents with $q_{Ibatt}=1$ and $q_{ISC}$ = 1. (b) A dissected view of the individual current.

Fig 16 shows the current plots for$\;{\tilde{I}}_{wave}$, $\;{\tilde{I}}_{gen}$, ${\tilde{I}}_{SC}$, and ${\tilde{I}}_{batt}$. In Fig 16a, we can see that the total current generated by ${\tilde{I}}_{gen}$, ${\tilde{I}}_{SC}$, and ${\tilde{I}}_{batt}$ effectively compensates for ${\tilde{I}}_{wave}$, with the current differences as shown in Fig 15. Fig 16b shows the individual currents graphs of ${\tilde{I}}_{gen}$, ${\tilde{I}}_{SC}$, and ${\tilde{I}}_{batt}$. As the weights increase, the difference between the battery current and the SC current also increases. Similar to the previous two wave profiles, the current differences begin to taper off when $q_{Ibatt}$ exceeds 20.

b)
$q_{Ibatt}=30$

**Fig 17:**
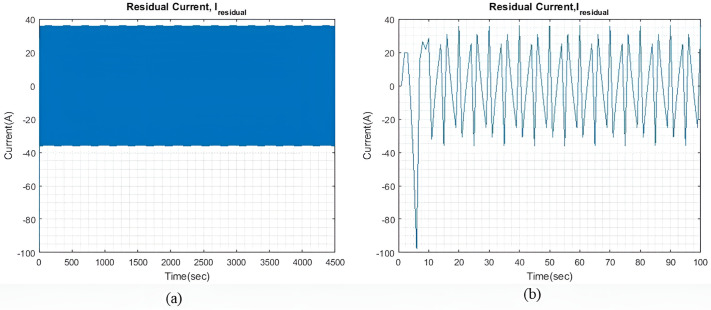
(a) ${\tilde{I}}_{residual\;}$ with $q_{Ibatt}=30$ and $q_{ISC}$ = 1. (b) Enhanced view of the ${\tilde{I}}_{residual\;}$ initial transient response.

Fig 17 shows the ${\tilde{I}}_{residual\;}$ when $q_{Ibatt}$ is increased to 30. It is noted that the transient response is significantly much larger when $q_{Ibatt}=$1, as shown in Fig 15. Throughout the simulation, oscillations of ±36A are maintained and the ${\tilde{I}}_{residual\;}$ is able to reach a steady state.

**Fig 18:**
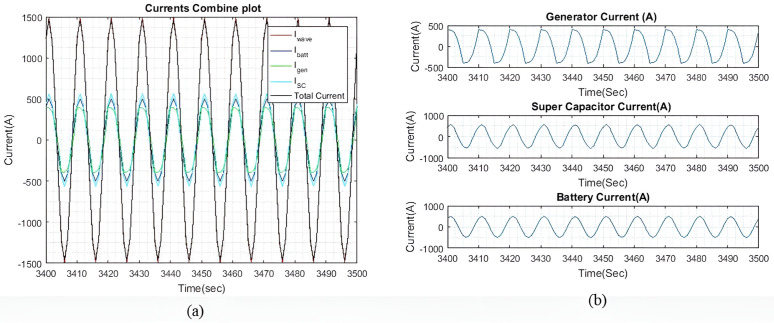
(a) Enhanced view of the ${\tilde{I}}_{gen}$, ${\tilde{I}}_{SC}$, and ${\tilde{I}}_{batt}$ output currents with $q_{Ibatt}=30$ and $q_{ISC}$ = 1. (b) A dissected view of the individual current.

Fig 18 shows the current plots for ${\tilde{I}}_{wave}$, $\;{\tilde{I}}_{gen}$, ${\tilde{I}}_{SC}$, and ${\tilde{I}}_{batt}$ when $q_{Ibatt}=30.$ Similar to the previous two wave profiles, as the weights increase, the difference between the battery current and the SC current also increases. The current difference between the battery and the SC begins to taper off when $q_{Ibatt}$ exceeds 20, while the ${\tilde{I}}_{gen}$, is maintained at 398A. Despite a higher ${\Delta I}_{gen}$, the ESDs are still capable of generating most of the required current to compensate for the wave current.

Table 6 shows the steady state peak output current for ${\tilde{I}}_{gen}$, ${\tilde{I}}_{SC}$, and ${\tilde{I}}_{batt}$ based on $q_{Ibatt}$. The difference in currents is not as significant as it was under moderate sea conditions due to the increased ${\Delta I}_{gen}$.

**Table 6:** Peak output currents for 45% wave profile.

$q_{Ibatt}$	${\tilde{I}}_{gen}$ (Amp)	${\tilde{I}}_{batt}$ (Amp)	${\tilde{I}}_{SC}$ (Amp)	${\tilde{I}}_{wave}$ (Amp)	${\tilde{I}}_{residual}$ (Amp)	Total Current (Amp)
1	398.2	534.8	535.2	1492	± 34.7	1465
5	398.4	513.6	553.7	1492	± 36	1462
10	398.3	507.1	559.7	1492	± 36	1462
20	398.2	502.9	563.7	1492	± 36	1462
30	398.1	501.9	565.3	1492	± 36	1462
50	398.1	499.8	566.7	1492	± 36	1461

One common observation in all three types of waveform is that SC and battery output current start to decline when the value of $q_{Ibatt}$ is above 20. This shows that due to increase in the difference between states more than 20 does not have a significant impact for this model. Thus, the HESS efficiently contributed in lowering the load on the generator and for the compensation of ${\tilde{I}}_{wave}$. Further, the MPC controller is implemented with a prediction horizon of 10 and a control horizon of 2. The sampling time is set to 0.1 seconds, enabling fast and responsive control suitable for dynamic conditions. The analysis highlights that while MPC involves solving an optimization problem using a quadratic programming (QP) solver at each control step, its real-time implementation is achievable using modern embedded processors and efficient solvers. By optimizing the prediction horizon and control update rates, computational demands can be managed without compromising performance. Additionally, employing fast optimization algorithms reduces latency, ensuring MPC remains a viable and effective solution for ship energy management under varying sea conditions.

D **Performance Analysis of State of Charges (SOC)**

**Fig 19:**
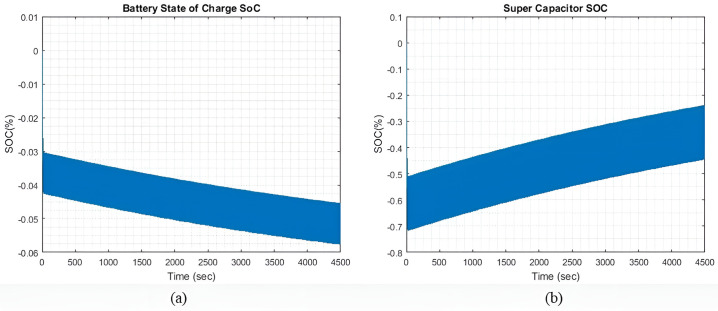
(a) Battery SoC. (b) SC’s SoC when $q_{Ibatt}=1$.

**Fig 20:**
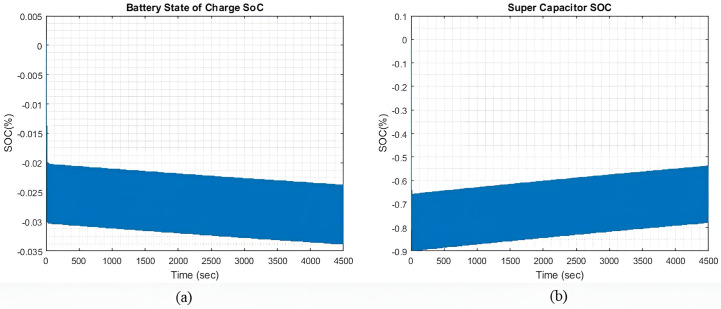
(a) Battery SoC. (b) SC’s SoC when $q_{Ibatt}=30$.

Figs 19 and 20 compare the SC and battery SOC at two different values of $q_{Ibatt}$ weight ($q_{Ibatt}=1$and $q_{Ibatt}=30$) respectively. The most commonly used scenario during sailing is the moderate sea wave profile condition. It appears that SC SOC attains its maximum rated value more rapidly at a lower $q_{Ibatt}$ than at a higher $q_{Ibatt}$ weightage. This is anticipated although both ESDs are providing almost equal amounts of current, while when the SC has attained its rated capacity, the extra current induced during the charging phase is shifted to charge the battery. When the SC SOC reaches the maximum limit as set in (24), the battery SOC begins to increase. When $q_{Ibatt\;}$ increases, the SC provides more current than the battery. The SC takes a longer time to achieve its maximum rated SOC, before the extra current gets switched to charge the battery.

Through our analysis, we quantify that increasing the battery weighting factor $q_{Ibatt}$ from 1 to 30 enhances overall energy storage capacity by reducing the battery’s current contribution by up to 60%, thereby shifting transient loads to the supercapacitor. However, this also results in a slight efficiency drop, as indicated by an increase in residual current from ±13 A to ±16 A in light sea conditions. Supercapacitors significantly improve transient performance, handling peak currents up to 554 A compared to 500 A for batteries during heavy sea conditions and reducing system response time from 50 seconds to approximately 35 seconds. This redistribution lowers battery State of Charge (SOC) variation by 15–20%, maintaining it within a safe 20–80% range, which helps extend battery lifespan. Across varying sea conditions (15%, 25%, and 45% wave profiles), the system maintains stable operation for 8 hours with generator current stabilized at 108 A, confirming improved energy efficiency, reduced degradation, and enhanced system reliability.

## 4. Conclusion

This paper investigates a hybrid ship energy management system using MPC to ensure smooth sailing under varying sea conditions. The ship’s generator faces substantial loading stress due to power fluctuations during navigation, especially in adverse conditions, potentially causing damage or failure. Although preliminary work is based on idealized and simplified scenarios, it shows that a control system uses the Francis Equation to model periodic perturbations, and MPC to address physical constraints, as a robust solution to this problem. The performance of HESS is assessed across different battery weight configurations, ranging from 1 to 50, and examines the effects of various combinations of battery and SC weights on system behavior. Notably, simulation results reveal that when the weightage difference among these energy storage components exceeds 20, saturation occurs in their respective currents. Simulations conducted under different sea conditions, including light, moderate, and heavy sea, convincingly demonstrate the effectiveness of the proposed methodology in mitigating power variations and preventing generator failures, ultimately research enhances ship reliability and environmental sustainability in adverse maritime conditions.

Going beyond simulation, the planned future work includes experimental validation through hardware-in-the-loop (HIL) testing and real-time implementation on maritime platforms to assess robustness under real-world conditions. Additional potential enhancements could integrate machine learning algorithms for predictive energy management, dynamically adapting MPC constraints based on real-time sea state estimations, and incorporating AI-driven autonomous control to further enhance system efficiency and adaptability. These advancements together with practical feasibility considerations will further strengthen the study’s impact by ensuring scalability and resilience of hybrid ship energy management strategies and will lead to sustainable maritime operations.

## References

[1] Guo J, Huang Q, Cui L. The impact of the Sino-US trade conflict on global shipping carbon emissions. J Cleaner Production. 2021;316:128381. doi: 10.1016/j.jclepro.2021.128381

[2] Wasim M, Ali A, Choudhry MA, Saleem F, Shaikh IUH, Iqbal J. Unscented Kalman filter for airship model uncertainties and wind disturbance estimation. PLoS One. 2021;16(11):e0257849. doi: 10.1371/journal.pone.0257849 34739486 PMC8570505

[3] Wang Q, Yang X. Imbalance of carbon embodied in South-South trade: evidence from China-India trade. Sci Total Environ. 2020;707:134473. doi: 10.1016/j.scitotenv.2019.134473 31863995

[4] International Maritime Organization. Review of Maritime Transport 2015. Geneva: United Nations; 2015. http://unctad.org/en/pages/PublicationWebflyer.aspx?publicationid=1374

[5] Yichao T, Khaligh A. On the feasibility of hybrid Battery/Ultracapacitor Energy Storage Systems for next generation shipboard power systems. In: 2010 IEEE Vehicle Power and Propulsion Conference. 2010: 1–6. doi: 10.1109/vppc.2010.5729211

[6] Ovrum E, Bergh TF. Modelling lithium-ion battery hybrid ship crane operation. Appl Energy. 2015;152:162–72. doi: 10.1016/j.apenergy.2015.01.066

[7] Geertsma RD, Negenborn RR, Visser K, Hopman JJ. Design and control of hybrid power and propulsion systems for smart ships: a review of developments. Appl Energy. 2017;194:30–54. doi: 10.1016/j.apenergy.2017.02.060

[8] Ali A, Söffker D. Towards optimal power management of hybrid electric vehicles in real-time: a review on methods, challenges, and state-of-the-art solutions. Energies. 2018;11(3):476. doi: 10.3390/en11030476

[9] Xie P, Tan S, Guerrero JM, Vasquez JC. MPC-informed ECMS based real-time power management strategy for hybrid electric ship. Energy Reports. 2021;7:126–33. doi: 10.1016/j.egyr.2021.02.013

[10] Luo Y, Fang S, Niu T, Chen G, Liao R. Power-characterized shipboard hybrid energy storage system management for dynamic positioning. Ocean Eng. 2024;298:117256. doi: 10.1016/j.oceaneng.2024.117256

[11] Tummuru NR, Mishra MK, Srinivas S. Dynamic energy management of renewable grid integrated hybrid energy storage system. IEEE Trans Ind Electron. 2015;62(12):7728–37. doi: 10.1109/tie.2015.2455063

[12] Raboaca MS, Bizon N, Grosu OV. Energy management strategies for hybrid electric vehicles - vosviwer bibliometric analysis. In: 2020 12th International Conference on Electronics, Computers and Artificial Intelligence (ECAI). 2020: 1–8. doi: 10.1109/ecai50035.2020.9223123

[13] Gautam AK, Tariq M, Pandey JP, Verma KS, Urooj S. Hybrid sources powered electric vehicle configuration and integrated optimal power management strategy. IEEE Access. 2022;10:121684–711. doi: 10.1109/access.2022.3217771

[14] Bo TI, Johansen TA. Battery power smoothing control in a marine electric power plant using nonlinear model predictive control. IEEE Trans Contr Syst Technol. 2017;25(4):1449–56. doi: 10.1109/tcst.2016.2601301

[15] Hou J, Sun J, Hofmann HF. Mitigating power fluctuations in electric ship propulsion with hybrid energy storage system: design and analysis. IEEE J Oceanic Eng. 2018;43(1):93–107. doi: 10.1109/joe.2017.2674878

[16] Øveraas H, Halvorsen HS, Landstad O, Smines V, Johansen TA. Dynamic positioning using model predictive control with short-term wave prediction. IEEE J Oceanic Eng. 2023;48(4):1065–77. doi: 10.1109/joe.2023.3288969

[17] Wang L. Model predictive control system design and implementation using MATLAB. London, UK: Springer; 2009.

[18] Ali SU, Waqar A, Aamir M, Qaisar SM, Iqbal J. Model predictive control of consensus-based energy management system for DC microgrid. PLoS ONE. 2023;18(1):e0278110. 10.1371/journal.pone.0278110 PMC9858890 36662901

[19] Ullah MI, Ajwad SA, Irfan M, Iqbal J. MPC and H-infinity based feedback control of non-linear robotic manipulator. In: 2016 International Conference on Frontiers of Information Technology (FIT). 2016: 136–41. doi: 10.1109/fit.2016.033

[20] Aro K, Urvina R, Deniz NN, Menendez O, Iqbal J, Prado A. A nonlinear model predictive controller for trajectory planning of skid-steer mobile robots in agricultural environments. In: 2023 IEEE Conference on AgriFood Electronics (CAFE). 2023: 65–9. doi: 10.1109/cafe58535.2023.10291643

[21] Maxwell T. 125 Volt Transportation Modules Ultracapacitors. 2020 [Accessed 2020 January 18]. https://www.maxwell.com/products/ultracapacitors/125v-tran-modules

[22] U.S. Naval Academy. Chapter 7 resistance and powering of ships. Class Notes for EN 400: Principles of Ship Performance. Annapolis, MD, USA: U.S. Naval Academy: 46. https://www.usna.edu/NAOE/_files/documents/Courses/EN400/02.07%20Chapter%207.pdf

[23] Alafnan H, Zhang M, Yuan W, Zhu J, Li J, Elshiekh M, et al. Stability improvement of DC power systems in an all-electric ship using hybrid SMES/battery. IEEE Trans Appl Supercond. 2018;28(3):1–6. doi: 10.1109/tasc.2018.2794472

[24] MATLAB. Solve a quadratic programming problem using the KWIK algorithm – MATLABmpcqpsolver. Accessed 2020 February. https://www.mathworks.com/help/mpc/ref/mpcqpsolver.html#d117e14529

[25] Aguilar CO, Krener AJ. Model predictive regulation. 2013 October 26. doi: ArXiv13107135Math

